# Mitochondrial Quality Control Mechanisms during Diabetic Cardiomyopathy

**DOI:** 10.31662/jmaj.2022-0155

**Published:** 2022-09-30

**Authors:** Melis Ketenci, Daniela Zablocki, Junichi Sadoshima

**Affiliations:** 1 Rutgers New Jersey Medical School, Department of Cell Biology and Molecular Medicine, Rutgers Biomedical and Health Sciences, Newark, USA

**Keywords:** Mitophagy, Autophagy, Diabetic Cardiomyopathy, Mitochondria, Diabetes Mellitus, Heart Failure

## Abstract

One of the major complications of diabetes mellitus is diabetic cardiomyopathy. One of the mechanisms that initiates the irreversible deterioration of cardiac function in diabetic cardiomyopathy is mitochondrial dysfunction. Functionally impaired mitochondria result in greater levels of oxidative stress and lipotoxicity, both of which exacerbate mitochondrial damage. Mitochondrial health is constantly monitored by mitochondrial quality control mechanisms. Mitophagy selectively degrades damaged mitochondria, thereby maintaining the healthy pool of mitochondria and preserving myocardial function. Mitophagy in diabetic cardiomyopathy is mediated by multiple mechanisms in a time-dependent manner. Potential targets for the treatment of diabetic cardiomyopathy include increased oxidative stress, mitochondrial dynamics, and mitochondrial clearance. Thus, stimulation of mitophagy represents a promising strategy for the alleviation of diabetic cardiomyopathy.

## Introduction

The incidence of diabetes has increased dramatically over the past three decades ^(1)^. Major triggers of diabetes include a sedentary lifestyle, obesity, and dyslipidemia ^(2)^. More than half of patients with diabetes develop cardiomyopathy, characterized by the presence of hypertrophy, diastolic dysfunction, and arrhythmia, which is consistently accompanied by mitochondrial dysfunction at the cardiomyocyte level ^(3)^. Mitochondria occupy approximately 30% of the cardiomyocyte volume and supply large amounts of ATP to support the energy demands of contraction. The presence of dysfunctional mitochondria in cardiomyocytes leads to increased oxidative stress and triggers a vicious cycle of mitochondrial damage and production of reactive oxygen species (ROS) ^(4)^. Mitochondrial health is maintained by various regulatory mechanisms, including mitochondrial fusion and fission, mitophagy, and biogenesis ^(5)^. Mitophagy is a major mechanism of degradation by which damaged mitochondria are selectively sequestrated in double-membraned vesicles called autophagosomes and subsequently eliminated by lysosomes ^(6)^. Our goal in this review is to summarize the molecular mechanisms of mitophagy and discuss rational therapeutic considerations for improving the quality of mitochondria and alleviating cardiac complications in patients with diabetes.

## Mitochondria and Diabetic Cardiomyopathy

Although type 1 and type 2 diabetes have different underlying mechanisms, both lead to similar detrimental cardiovascular outcomes. Cardiovascular complications in diabetes are associated with high morbidity and mortality rates and are caused by accelerated atherosclerosis, coronary artery disease, myocardial infarction, and heart failure ^(1)^. Diabetic cardiomyopathy was first described in 1972 and was later accepted as a distinct clinical entity in the 1974 Framingham Heart Study ^(7), (8)^. It is defined as a cardiac dysfunction in the presence of diabetes mellitus that is not caused by ischemic, valvular, or hypertensive heart disease. Other types of cardiomyopathies, including hypertrophic, restrictive, and dilated cardiomyopathies, are also excluded as potential causes of the dysfunction ^(9)^. In the early stage, diabetes causes diastolic dysfunction and heart failure with preserved ejection fraction, but it may later lead to systolic dysfunction, called heart failure with reduced ejection fraction, in the chronic phase ^(2), (10)^. Other structural and functional changes observed in the heart during diabetes include ventricular hypertrophy, cardiac steatosis, and cardiac arrhythmia ^(3)^.

The adult heart is a high energy demand organ and continuously produces a large amount of ATP, using fatty acid as the major source of fuel (60% of the ATP produced) ^(11), (12)^. However, diabetic hearts lack metabolic flexibility, depending on fatty acid oxidation more critically than healthy normal hearts because of the impaired uptake and utilization of glucose due to insulin deficiency or resistance ^(13), (14)^. In healthy cardiomyocytes, the low levels of electron leakage from the electron transport chain at the time of oxidative phosphorylation can be cleared by antioxidants, such as glutathione reductase, glutathione peroxidase, catalase, and superoxide dismutase (SOD). However, in diabetic hearts, greater amounts of ROS are produced as a result of increased levels of fatty acid oxidation, exceeding the cells’ capacity to neutralize oxidants. Mitochondrial proteins, including those in the electron transport chain, are damaged by oxidative stress and, in turn, produce more ROS ^(15)^. In addition, the NADPH oxidase, Nox4, is an important source of ROS in hyperglycemia, and its upregulation may be involved in early structural changes seen in diabetic cardiomyopathy caused by type 1 diabetes ^(16)^. Increased calcium influx into mitochondria from the endoplasmic reticulum has detrimental effects on both mitochondrial and cardiac functions in diabetes ^(17)^. Damaged and futile mitochondria undergo fragmentation, separation, and elimination. However, as we will discuss later, they can accumulate if the clearance mechanism is insufficient, leading to the production of more ROS, which, in turn, exacerbate mitochondrial dysfunction ^(18), (19)^. Mitochondrial dysfunction also leads to the attenuation of fatty acid oxidation. An imbalance among fatty acid intake, lipolysis, and fatty acid oxidation leads to the accumulation of cytotoxic lipids, including ceramides and diacylglycerols, a condition termed lipotoxicity ^(20)^. Mitochondrial dysfunction, energetic insufficiency, and lipotoxicity all contribute to impaired cardiac function in diabetic cardiomyopathy ^(21), (22)^. Diabetic cardiomyopathy is also accompanied by inflammation and cellular senescence, but their involvement is not discussed in this article. (The readers are referred to an excellent review of the involvement of inflammation and cellular senescence in the development of diabetic cardiomyopathy ^(23)^).

## Mitochondrial Quality Control Mechanisms

**Figure 1.**
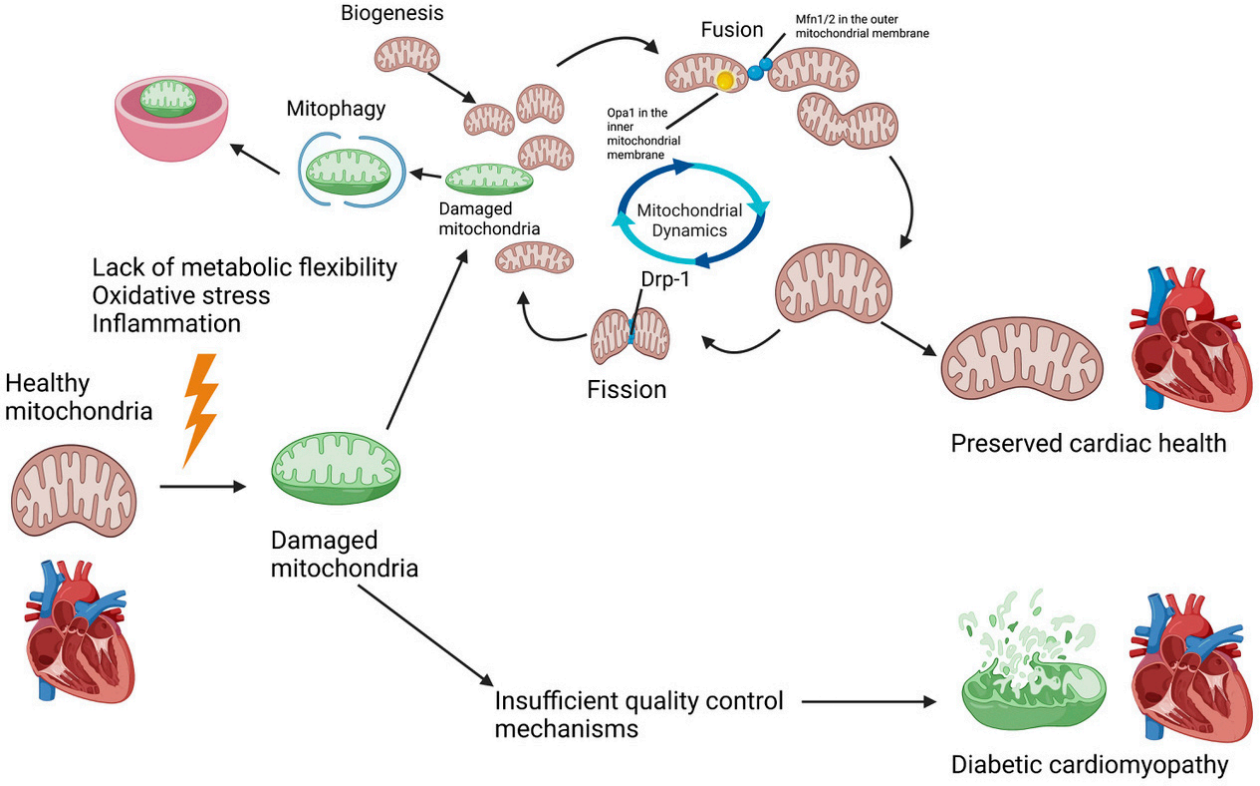
The involvement of mitochondrial dysfunction in the pathogenesis of diabetic cardiomyopathy. Although the activation of the mitochondrial quality control mechanism mitigates mitochondrial dysfunction and the development of diabetic cardiomyopathy, insufficient activation leads to exacerbation of mitochondrial dysfunction and diabetic cardiomyopathy. This figure was created with a software provided at BioRender.com.

Damaged mitochondria not only are dysfunctional but can also be toxic since they exacerbate ROS production, release cell death-promoting factors, and induce mutations in mitochondrial DNA. Thus, it is critical for the cell to monitor the quality of mitochondria and expeditiously eliminate those that are dysfunctional. Mitochondrial quantity, quality, and morphology are under constant regulation by several different mechanisms. These include mitochondrial dynamics (i.e., fission and fusion), mitophagy, and mitochondrial biogenesis (Figure 1) ^(5)^.

Mitochondria are dynamic organelles that frequently change shape, size, and quantity. Damaged mitochondria lose their membrane potential, which is the driving force for ATP production, and this occurs even more rapidly in diabetic hearts ^(24)^. In mitochondrial fusion, depolarized mitochondria merge with others, allowing each mitochondrion to compensate for its own defects by mixing with new constituents to complement its own mtDNA. It is generally believed that fusion is a useful mechanism that increases cellular viability ^(5)^. In contrast, mitochondrial fission involves asymmetric segregation ^(25)^ of damaged mitochondria that occurs at sites of contact between mitochondria and the endoplasmic reticulum via recruitment of Drp1. In general, mitochondrial fission serves to prepare the cell for subsequent mitophagy ^(26)^. Fusion and fission are delicately balanced with each other, and tipping the scale in favor of either one can become detrimental to the cell ^(27)^. Mitochondrial fusion is regulated by Mfn1 and Mfn2 on the outer mitochondrial membrane and Opa1 on the inner mitochondrial membrane. Regulators of mitochondrial fission include Drp1, FIS1, Mff, and MiD49/51. Drp1 translocates from the cytosol to mitochondrial fission sites with the help of other fission proteins ^(5), (28)^. There are also special mitochondria-endoplasmic reticulum contact sites, termed mitochondria-associated membranes. These are involved in mitochondrial fission and the regulation of mitochondrial calcium balance and oxidative stress ^(29)^. Mfn2 and FUNDC1 are present at these contact sites and are involved in the regulation of the Ca^2+^ homeostasis of mitochondria and endoplasmic reticulum. Inhibition of Mfn2 decreases significant mitochondrial calcium overload and oxidative stress and improves both cardiac systolic and diastolic dysfunction under hyperglycemic conditions. FUNDC1 on these contact sites also has an important function, and its deletion causes impaired mitochondrial fission with larger mitochondria dominating in the cell. Although this could be perceived as a protective mechanism for the heart, loss of FUNDC1 results in mitochondria with decreased membrane potential, leading to diastolic and systolic dysfunction with fibrosis ^(30)^.

The role of mitochondrial fusion and fission in the heart appears complex and context dependent. For example, inhibition of Drp1 with Mdvi-1 increases fused mitochondria and improves cardiac function during doxorubicin treatment ^(31)^. However, downregulation of Drp1 in cardiomyocytes below baseline leads to dysfunctional mitochondria with decreased mitochondrial membrane potential, increased opening of the mitochondrial permeability transition pore, and compromised ATP production ^(32)^. In addition, increased mitochondrial fission during hyperglycemia protects mitochondrial quality since inhibiting fission increases cell death, whereas Drp1 overexpression prevents high glucose-induced cardiotoxicity ^(33)^. These studies all suggest that the delicate balance of fusion and fission relative to one another is an essential part of maintaining mitochondrial quality in diabetic hearts ^(34)^.

## Autophagy and Mitophagy

The term autophagy refers to the mechanisms by which a cell “eats itself,” degrading cellular materials to maintain cellular homeostasis ^(35)^. Autophagy occurs via macroautophagy, microautophagy, and chaperone-mediated autophagy. In macroautophagy (hereafter autophagy), cellular constituents are delivered to lysosomes in double-membraned autophagic vesicles that are formed via the well-coordinated actions of autophagy-related (Atg) genes. In contrast, in microautophagy, lysosomes directly engulf dysfunctional and damaged organelles, and chaperone-mediated autophagy involves lysosomal sequestration of cellular constituents with the help of chaperones that tag specific KFERQ sequences of target proteins ^(36)^. General autophagy occurs via multiple steps. AMPK, the energy sensor of the cell, detects decreased energy/ATP levels in the cell and initiates autophagy by phosphorylating Ulk1. Autophagy then begins with the formation of a layer of membrane from the endoplasmic reticulum membrane and continues via the formation of multiple complexes that activate one another, including Beclin1/Vps34/Vps15, involved in nucleation, and LC3-Atg12/5, involved in elongation. Finally, the double-membraned autophagic vesicle maturates and closes with the incorporation of LC3-II. Autophagosomes then fuse with lysosomes for final degradation of the engulfed cellular components ^(37)^.

The high levels of autophagy seen during the first few hours of life, when nutrient availability is relatively low, demonstrate a highly adaptive aspect of this mechanism ^(12)^. Autophagy either can occur non-selectively or can target particular organelles, including mitochondria and peroxisomes. When autophagy selectively targets mitochondria, it is called mitophagy ^(38)^. Autophagy is activated or deactivated in accordance with the type of stress ^(39)^, and the direction of the change in autophagy can have different consequences in different pathologies, ranging from cellular protection to cellular death ^(40)^. At the time of an initial insult to the organism, as in consumption of a high-fat diet (HFD) by mice, autophagic flux generally increases ^(41)^. However, the activation of autophagy is often transient, and the subsequent inactivation may prevent the organism from being able to cope with the pathologic stress, thereby leading to the development or deterioration of the disease process ^(42)^.

Mitophagy is a mitochondria-specific form of autophagy that selectively degrades dysfunctional/damaged or unnecessary mitochondria to prevent their harmful effects or accumulation in the cell ^(27), (43), (44), (45)^. One of the most well-studied mechanisms of mitophagy occurs via the PINK1-Parkin pathway. Mutations in PINK1 and Parkin are involved in the pathogenesis of early-onset Parkinson’s disease ^(46), (47)^. PINK1 is a serine-threonine kinase that exists in two forms and resides in two subcellular compartments. Mitochondrial PINK1 is the longer form, accumulating specifically on the membrane of damaged and depolarized mitochondria. Presenilin-associated rhomboid-like protein, a protease, cleaves the long form of PINK1, thereby turning it into the shorter cytosolic form and preventing its accumulation on healthy, functioning mitochondria with a high (polarized) membrane potential ^(34), (48)^. PINK1 stabilization and phosphorylation of the ubiquitin ligase, Parkin, allow the activation of Parkin at the outer mitochondrial membrane. Although PINK1 accumulation triggers the acceleration of Parkin recruitment to mitochondria, a previous study found that the actions of PINK1 may not be essential, and that mitophagy may ensue in the presence of Parkin alone ^(49)^. After Parkin is recruited to mitochondria, it tags damaged mitochondrial proteins via ubiquitination. Ubiquitinated proteins are then bound by adapter proteins possessing both a ubiquitin binding domain and LC3 interacting regions (LIRs), including p62, optineurin, and NDP52. After separation of the damaged portion from the healthy area through either Drp1-dependent or -independent mechanisms, autophagosomes carrying mitochondria merge with lysosomes for degradation. In another form of mitophagy, proteins harboring LIRs, including FUNDC1, BNIP3, and NIX/BNIP3L, expressed on the damaged mitochondria are recognized by LC3 ^(15)^. Growing lines of evidence suggest that degradation of damaged mitochondria is also mediated through a mechanism, termed alternative mitophagy, that is independent of the conventional machinery, including Atgs and LC3. Although the functional significance of the alternative mechanism of mitophagy remains unclear, it appears to act as a compensatory mechanism when conventional mechanisms of mitophagy are downregulated, as in chronically pathologic conditions in vivo.

Degraded mitochondria are replaced with new mitochondria through mitochondrial biogenesis. Mitophagy and mitochondrial biogenesis coordinately regulate mitochondrial content to maintain energy metabolism. For example, Parkin-induced degradation of Paris may stimulate mitochondrial biogenesis by alleviating Paris-induced suppression of PGC-1α, a transcription coactivator and the master regulator of mitochondrial biogenesis ^(50)^.

## Mitophagy in Diabetic Cardiomyopathy

**Figure 2.**
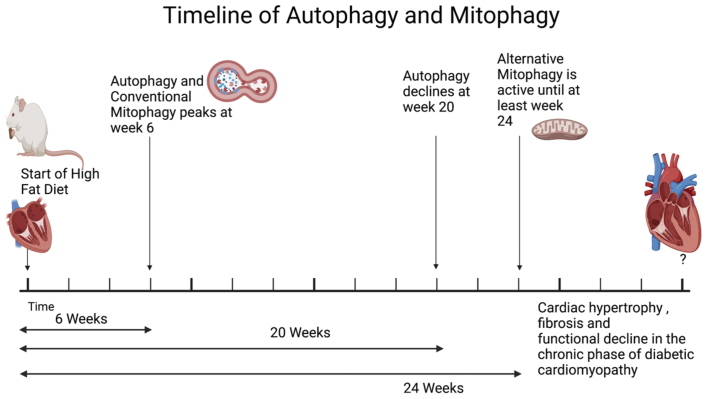
A proposed time course depicting how autophagy and mitophagy are regulated during the initial development of cardiomyopathy in response to high-fat diet (HFD) consumption in a mouse model of obesity-induced cardiomyopathy. Mitophagy is rapidly activated by HFD consumption through the Atg7- and LC3-dependent mechanism commonly used in general autophagy. The initial activation of autophagy/mitophagy is inactivated by 20 weeks of HFD consumption. However, mitophagy continues to function through alternative mitophagy, which is an Atg7- and LC3-independent but Ulk1- and Rab9-dependent mechanism, during the chronic phase of HFD consumption. The activation of mitophagy is essential for the maintenance of mitochondrial quality and cardiac function in hearts with obesity-induced cardiomyopathy. This figure was adapted from Experimental Timeline (Horizontal, Black and White) by BioRender.com at https://app.biorender.com/biorender-templates.

Although the activation of autophagy and mitophagy has been demonstrated in diabetic hearts, the extent of mitophagy activation and the underlying molecular mechanisms appear to be time dependent. In a mouse model of obesity-induced cardiomyopathy, autophagy is activated in the heart in response to HFD (60% kcals from fat) consumption, peaking at 6 weeks (Figure 2) ^(22)^. During the early phase of HFD consumption, the majority of autophagosomes in the heart are colocalized with mitochondria, and mitophagy is activated through a Parkin-dependent mechanism. This activation of mitophagy is attenuated in cardiac-specific *Atg7* knockout (*Atg7* cKO) mice, which are autophagy-deficient, suggesting that mitophagy during the early phase of HFD consumption utilizes the mechanism commonly used in general autophagy ^(22)^. *Atg7* cKO mice exhibit more severe mitochondrial dysfunction and cardiomyopathy than wild type mice, suggesting that the heart activates autophagy to stimulate mitophagy, which plays an important role in eliminating damaged mitochondria and maintaining cardiac function during the acute phase of HFD consumption. In another study, the development of cardiomyopathy after 4 months of HFD consumption was similarly attenuated in PINK1 knockout mice ^(51)^. It should be noted that, although the mouse heart exhibits signs of diastolic dysfunction 1 month after HFD consumption, other morphological features of cardiomyopathy, including hypertrophy, steatosis, inflammation, and fibrosis, are not obvious until after 3 months of HFD consumption. This activation of mitophagy during the acute phase of HFD consumption is an important defense mechanism against cardiac remodeling and cardiac dysfunction; fully established features of cardiomyopathy are manifested during the acute phase of HFD consumption only when the activation of mitophagy is inhibited ^(22)^. This suggests that early intervention to maintain mitochondrial quality is essential to delaying the progression of cardiomyopathy.

Despite its critical importance, the activation of general autophagy and mitophagy is transient, and the level of autophagy and mitophagy declines to baseline by 20 weeks of HFD consumption ^(22)^. Inactivation of autophagy is commonly observed in many experimental models of diabetic cardiomyopathy. Although the mechanisms mediating the rapid inactivation of autophagy remain unclear, it has been shown that signaling mechanisms inhibiting autophagy, including one mediated by the serine-threonine kinase, Mst1, are activated ^(36)^. Further investigation is needed to elucidate the specific mechanisms by which autophagy is inactivated during the chronic phase of diabetic cardiomyopathy. Targeting these inhibitory mechanisms may also delay the progression of diabetic cardiomyopathy.

## Mitophagy in Chronic Diabetic Cardiomyopathy

Interestingly, although general autophagy is no longer active after 20 weeks of HFD consumption, mitophagy, primarily mediated through *alternative mitophagy*, continues to be observed even after general autophagy is inactivated ^(52)^. Since systolic cardiac dysfunction is not yet observed at this time point, alternative mitophagy presumably acts as a compensatory mechanism. Delayed activation of alternative mitophagy after inactivation of conventional mitophagy is also observed in the heart during acute pressure overload, suggesting that alternative mitophagy acts as a backup mechanism to delay the development of mitochondrial dysfunction in response to stress. Mitophagy activated during the chronic phase of HFD consumption is mediated by an Ulk1- and Rab9-dependent but Atg7- and Parkin-independent mechanism, consistent with the aforementioned alternative mitophagy. Suppression of alternative mitophagy in Rab9 S179A knock-in or cardiac-specific Ulk1 knockout mice exacerbated the development of mitochondrial dysfunction and cardiomyopathy ^(52)^. Thus, regardless of the underlying mechanisms involved, mitophagy activation appears critical for the maintenance of mitochondrial function in both the acute and chronic phases of obesity and diabetic cardiomyopathy. In fact, TAT-Beclin1 (TB1), a cell-permeable short peptide that potently induces autophagy and mitophagy by mobilizing endogenous Beclin1 from its intracellular storage site, can reactivate or enhance mitophagy under a wide variety of conditions, thereby alleviating obesity-induced cardiomyopathy ^(22)^. Compared to the wealth of knowledge about the molecular mechanism of the conventional form of mitophagy, such as the involvement of PINK1 and Parkin, the molecular mechanism of alternative mitophagy remains poorly understood. Alternative mitophagy is often observed after conventional mitophagy is activated. The accumulation of dysfunctional mitochondria as a result of inactivation of conventional mitophagy alerts the cell by stimulating signaling mechanisms, including TFE3, which in turn upregulates transcriptional mechanisms ^(52)^. Further investigation of the mechanisms modulating the transcriptome that orchestrates the initiation of alternative mitophagy may provide useful information for improving mitochondrial quality control during the chronic phase of diabetic cardiomyopathy.

## Management of Diabetic Cardiomyopathy

Currently, there is no effective treatment regimen to reverse or control the progression of diabetic cardiomyopathy. Strict glycemic control with prevention of other related complications is the most accepted initial approach. Nutrition is a cornerstone for successful management of the disease. Ketone ester supplementation has been shown to improve cardiac function in diabetic mice ^(53)^. Following diagnosis of diabetic cardiomyopathy, cardiac changes are rapidly progressive, and there is a high risk of progression to heart failure. Since mitochondrial dysfunction is the most common driver of diabetic cardiomyopathy, excess oxidative stress, lipotoxicity, mitochondrial dynamics, mitophagy, and mitochondrial calcium homeostasis all constitute potential therapeutic targets. A similar concept has been proposed for the treatment of neurodegenerative disease, where improving mitochondrial quality control mechanisms alleviates the disease progression ^(54)^. Some studies have used mitochondria-targeting agents to decrease the production of ROS and improve impaired mitochondrial function, which ultimately prevents undesirable cardiac outcomes even in other cardiac pathologies. Cardiac structural and functional changes and even vascular damage can be prevented by administration of the mitochondria-targeting antioxidant, MitoQ ^(55), (56)^. Another study demonstrated that treatment with MnSOD, an antioxidant, preserved mitochondria and cardiac structure in diabetic OVE26 mice, which are associated with diminished levels of MnSOD ^(19), (57)^. However, despite the promising results of antioxidant therapy in animal studies, its use has not been proven to be beneficial in humans ^(58)^. Manipulation of mitophagy represents another possible approach to prevent mitochondrial dysfunction. Several agents target the different steps of mitophagy. Intraperitoneal injection of TB1 into mice increases LC3-II primarily in the mitochondrial fraction and reverses functional changes associated with diabetic cardiomyopathy. TB1 mobilizes endogenous Beclin1 from its intracellular anchorage site and stimulates mitophagy, the level of which is insufficient for the maintenance of mitochondrial function during the chronic phase of diabetic cardiomyopathy ^(22)^. High expression of the transcriptional and epigenetic regulator, Bromodomain-containing protein 4 (BRD4), during HFD consumption can be inhibited by JQ1, a chemical inhibitor of BRD4, resulting in increased PINK1-Parkin-dependent mitophagy and alleviation of the structural and functional changes associated with diabetic cardiomyopathy ^(51)^. Melatonin, a natural regulator of the sleep-wake cycle ^(59)^, accumulates in mitochondria and increases PINK1-Parkin-mediated mitophagy in diabetic hearts ^(60)^. Metformin also upregulates autophagy through its effects on AMPK and Beclin1 and preserves cardiac function in diabetic cardiomyopathy ^(61)^. Mst1, a serine-threonine kinase and a component of the Hippo pathway, inhibits Parkin-mediated mitophagy and causes the accumulation of dysfunctional mitochondria. Thus, a small molecule inhibitor of Mst1 may rescue depressed autophagy and improve both diastolic and systolic functions ^(62), (63)^. SGLT-2 inhibitors, highly effective antidiabetic agents, prevent cardiovascular complications of diabetes mellitus type 2 ^(64)^. The dual inhibitor sotagliflozin and SGLT-2 inhibitor empagliflozin decrease cardiac remodeling ^(65), (66)^. Empagliflozin improves diabetes-related microvascular injury through inhibition of mitochondrial fragmentation in cardiac microvascular endothelial cells, which results in decreased oxidative stress. It leads to improved cardiac nutrient and oxygen supply and increased resistance of cardiac microvascular cells to hyperglycemic insult ^(66)^. Of the many beneficial effects of SGLT-2 inhibitors, their effect on mitochondria appears particularly important. SGLT-2 inhibitors generally suppress mitochondrial fission by regulating the relative amounts of mitochondrial fusion-fission proteins, although some may exert their effects through stimulation of AMPK, with the same outcome ^(67)^. Ipragliflozin increases the level of mitochondrial fusion proteins and protects mitochondrial shape and structure in kidney proximal tubular epithelial cells ^(68)^. H_2_S, an endogenous gasotransmitter, decreases mitochondrial fragmentation by regulating fusion-fission proteins, but it can also improve mitophagy during hyperglycemia and hyperlipidemia ^(69)^. Small molecules identified through screening for the ability to induce mitochondrial fragmentation and autophagosome formation ameliorate proteostasis defects associated with neurodegenerative diseases in *Caenorhabditis elegans* in a Pink1-dependent manner ^(70)^. Thus, similar approaches may allow identification of modalities that would effectively protect mitochondria in the presence of diabetic cardiomyopathy.

## Conclusion and Future Perspectives

Diabetic cardiomyopathy is insidiously progressive. Unfortunately, there is currently no specific treatment to reverse or slow the pathogenesis of diabetic cardiomyopathy. As discussed in this review, however, inducing or enhancing the mitochondrial quality control mechanisms represents a reasonable approach to treat and potentially reverse the progression of diabetic cardiomyopathy. Nevertheless, many questions remain. First, how mitochondrial quality is affected and how autophagy/mitophagy is regulated at various stages of diabetic cardiomyopathy need to be clarified in the human heart. It would be useful to identify convenient biomarkers. Second, the endogenous mechanism by which general autophagy is inactivated after transient activation during the acute phase of cardiomyopathy needs to be clarified. This would provide hints to prolonging the endogenous mechanism of autophagy into the chronic phase of diabetic cardiomyopathy. Third, since alternative mitophagy is the predominant form of mitophagy during the chronic phase of diabetes, it can be a primary target in patients with chronic diabetes for longer term. Thus, the molecular mechanisms of alternative mitophagy, namely, how damaged mitochondria are identified and how autophagosomes are formed, need to be clarified. The endogenous (upstream) signaling mechanisms that stimulate alternative mitophagy also require further clarification. Finally, although induction of autophagy and mitophagy is generally protective, the optimal timing and extent of autophagy and mitophagy induction need to be explored carefully since inappropriate timing and/or intensity could exacerbate cell death and heart failure. By addressing these questions, it may be possible to identify a specific intervention to best preserve mitochondria in patients with diabetes and delay the development of diabetic cardiomyopathy.

## Article Information

### Conflicts of Interest

None

### Sources of Funding

This work was supported in part by the U.S. Public Health Service grant numbers HL67724, HL91469, HL102738, HL112330, HL138720, HL144626, HL150881, and AG23039 (J.S.), the American Heart Association Merit Award 20 Merit 35120374 (J.S.), and the Fondation Leducq Transatlantic Network of Excellence 15CVD04 (J.S.).

### Author Contributions

M.K., D.Z., and J.S. wrote the manuscript. The authors reviewed and approved the manuscript for publication.

### Disclaimer

Junichi Sadoshima is one of the Advisory Board members of JMA Journal and on the journal’s Editorial Staff. He was not involved in the editorial evaluation or decision to accept this article for publication at all.
